# Ticagrelor Enhances Release of Anti-Hypoxic Cardiac Progenitor Cell-Derived Exosomes Through Increasing Cell Proliferation *In Vitro*

**DOI:** 10.1038/s41598-020-59225-7

**Published:** 2020-02-12

**Authors:** Valentina Casieri, Marco Matteucci, Emilio M. Pasanisi, Angela Papa, Lucio Barile, Regina Fritsche-Danielson, Vincenzo Lionetti

**Affiliations:** 1 Institute of Life Sciences, Scuola Superiore Sant’Anna, Pisa, Italy; 2 Fondazione Toscana G. Monasterio, Pisa, Italy; 3 Laboratory for Cardiovascular Theranostics, Cardiocentro Ticino Foundation, 6900 Lugano, Switzerland; 4 Faculty of Biomedical Sciences, Università Svizzera Italiana, 6900 Lugano, Switzerland; 5 Research and early clinical development, Cardiovascular, Renal and Metabolism, Biopharmaceuticals R&D, AstraZeneca, Gothenburg, Sweden

**Keywords:** Cell biology, Molecular medicine

## Abstract

Despite the widespread clinical use of cardioprotection by long-term direct antagonism of P2Y12 receptor, underlying mechanisms are unclear. Here, we identify how release of pro-survival exosomes from human cardiac-derived mesenchymal progenitor cells (hCPCs) is regulated by clinically relevant dose of ticagrelor (1 μM), an oral selective and reversible non-thienopyridine P2Y_12_ inhibitor. Ticagrelor-induced enhancement of exosome levels is related to increased mitotic activity of hCPCs. We show a drug-response threshold above which the effects on hCPCs are lost due to higher dose of ticagrelor and larger adenosine levels. While it is known that pan-Aurora kinase inhibitor halts cell proliferation through dephosphorylation of histone H3 residue Ser10, we demonstrate that it also prevents ticagrelor-induced effects on release of cardiac progenitor cell-derived exosomes delivering anti-apoptotic HSP70. Indeed, sustained pre-treatment of cardiomyocytes with exosomes released from explant-derived hCPCs exposed to low-dose ticagrelor attenuated hypoxia-induced apoptosis through acute phosphorylation of ERK42/44. Our data indicate that ticagrelor can be leveraged to modulate release of anti-hypoxic exosomes from resident hCPCs.

## Introduction

Acute myocardial infarction (AMI) is an adverse cardiac event leading to high risk of morbidity and mortality worldwide^1^. Indeed, it has been demonstrated that a significant residual microvascular perfusion deficit remains after coronary revascularization^2^, which leads to cardiac cell death and progressive ventricular remodeling^3^. Post-ischemic left ventricular (LV) remodeling is a complex scenario mainly characterized by progressive loss of cardiomyocytes due to apoptosis and alteration of intercellular cross talk following prolonged exposure to hypoxic microenvironment^3^. In order to improve AMI care, adequate pharmacological approach to safely increase hypoxic tolerance of cardiomyocytes remains a critical need.

Numerous experimental^4–8^ and clinical^9–12^ studies have shown that long-term antagonism of P2Y_12_, a G protein-coupled (GPCR) purinergic receptor, protects the myocardium against ischemic LV remodeling. Chronic administration of ticagrelor, a selective and reversible P2Y_12_ receptor antagonist that does not require metabolic activation, is more cardioprotective than clopidogrel^13,14^, a prodrug that irreversibly inhibits P2Y_12_. The non-thienopyridine P2Y_12_ inhibitor such as ticagrelor enhances cardiomyocyte tolerance against ischemic microenvironment^4,7,8,15^ beyond its clinical efficacy in preventing intracoronary platelet aggregation. Although previous studies suggested that cardioprotective pleiotropic effects of ticagrelor are dependent on the enhanced levels of adenosine^16^, this mechanistic relationship was recently questioned by others^17^. In particular, recent study demonstrated that higher dose of ticagrelor (10 μM/L), which potentiates extracellular adenosine concentration compared to lower dose^18^, failed to protect ischemic reperfused rodent heart^19^. Although such a high dose of ticagrelor has not yet been tested in patients, we cannot exclude that chronic P2Y_12_ antagonism by lower dose of ticagrelor may lead to cardioprotective milieu in the myocardium through hitherto unexpected pleiotropic effects.

Cardiac-derived progenitor cells (CPCs), non-myocyte cells expressing mesenchymal/stromal cell surface markers even termed cardiac-derived mesenchymal progenitor cells, are involved in myocardial homeostasis after injury^20^. Indeed, these cells are able to release exosomes, smallest membrane-surrounded extracellular nanovesicles, which hamper cardiomyocyte apoptosis due to serum-nutrient starvation in a dose-dependent manner and attenuate LV remodeling in infarcted rodent heart^21–23^. Emerging evidences have also demonstrated that release of anti-apoptotic exosomes from explant-derived CPCs may be enhanced by microenvironment stimuli, such as hypoxia^24^. However, the study of the regulatory role of conventional cardiovascular drugs on release of human CPCs-derived exosomes is still at its infancy. Preliminary study demonstrated that P2Y_12_ antagonists decrease the release of pro-coagulant extracellular vesicles from activated platelets^25^, but the regulatory effects of ticagrelor on release of anti-apoptotic exosomes from adult cardiac-resident cells (i.e.: auricle-derived CPCs) has not yet been investigated. Since enrichment of plasma exosomes with those released from transplanted cardiac progenitor cells protected the ischemic myocardium^26^, we hypothesized that similar results might be obtained by pharmacological stimulation of cardiac-resident mesenchymal progenitor cells and without exosome transplantation. Since P2Y_12_ receptors are generally expressed in progenitor cells^27^, it is conceivable that long-term antagonism of P2Y_12_ receptor of human CPCs by ticagrelor may promote the release of exosomes protecting cardiomyocytes against hypoxia-induced apoptosis. For this purpose, we have used a well-characterized *in vitro* model of ischemic/hypoxic cardiomyocyte death based on murine HL1 cardiomyocytes chronically exposed to severe hypoxia^28^.

## Results

### Profile of adult explant-derived hCPCs

Cells were derived from human atrial appendage specimens cultured *ex vivo* as heart explants in accord with our previous studies^21,23^. Cells derived from auricle were a non-pure population. As shown in Supplemental Fig. 1, cells expressed mesenchymal/stromal markers (CD90, CD73, CD105), but not the common leukocyte antigen CD45. CD117 expression was almost absent. Since we have shown that atrial biopsy-derived cells express early cardiac markers (GATA4, MEF2c) and exhibit mesenchymal multilineage differentiation potential^21,23^, we term these cells human cardiac-derived mesenchymal progenitor cells, for which we used the acronym hCPCs for short.

### Long-term treatment with lower dose of ticagrelor increases exosome levels

**Figure 1:**
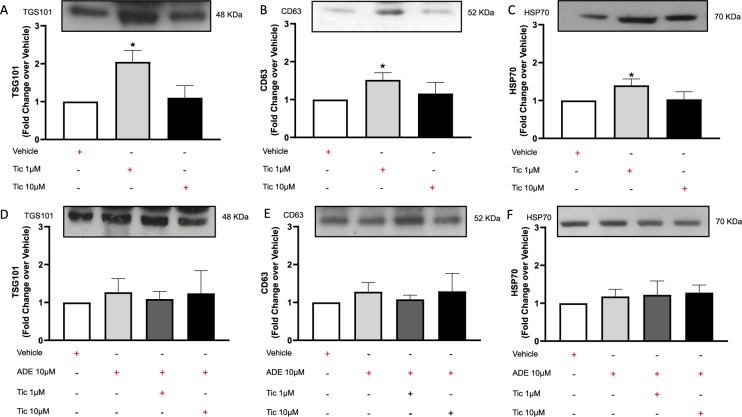
Long-term treatment for 72 h of human cardiac progenitor cells (hCPCs) with low-dose (1 μM) of ticagrelor (Tic) enhances the release of TSG101+ (**A**; 48KDa MW), CD63 + (**B**; 52KDa MW) and heat shock protein (HSP)-70 + (C; 70KDa MW) exosomes, but not at higher dose (Tic 10 μM). As shown in panels **D**–**F**, adenosine (ADE, 10 μM) *per se* does not enhance exosome release and its addiction to culture medium counteracts the effects of Tic 1 µM. Adenosine is added in the presence of EHNA (10 μM). Representative images of cropped densitometric bands of proteins TSG101, CD63 and HSP70 are showed in each panel. The full-length blots/gels of panels A–C and D–F are presented in Supplementary Figure 5 panel A and B respectively. All measurements are mean ± SD. *p < 0.05 vs. untreated condition (Vehicle: sterile phosphate buffer solution).

Adult hCPCs express P2Y_12_ receptors as for adult vascular smooth muscle cells (Supplemental Fig. 2). As shown in Fig. 1A–C, conditioned medium of proliferating hCPCs after treatment with lower dose of ticagrelor was enriched in vesicles expressing typical exosomal markers such as TSG101 + (A), CD63 + (B) and HSP70 + (C)^29^. Higher dose of ticagrelor did not increase exosome levels. Interestingly, effective dose of ticagrelor did not affect intracellular HSP70 levels (Supplemental Fig. 3). Exosome levels were not increased by long-term treatment of hCPCs with exogenous adenosine per se, which instead counteracted the effects of low-dose ticagrelor and did not enhance the effects of high-dose ticagrelor on extracellular exosome levels (Fig. 1D–F).

### Ticagrelor-induced Exosome release is related to higher hCPCs proliferation

**Figure 2:**
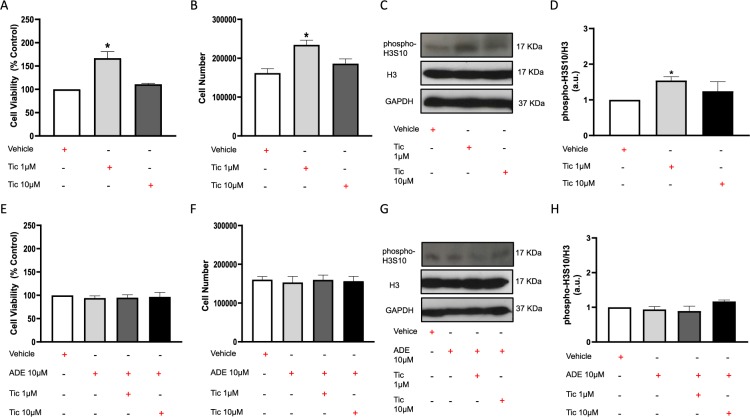
Long-term treatment for 72 h of human cardiac progenitor cells (hCPCs) with low-dose (1 μM) of ticagrelor (Tic) increases the cell viability (**A**), the cell number of viable cells (**B**) and the human phosphorylated Histone H3 on Serine 10 (phospho-H3S10; 17KDa MW)/total histone H3 (H3; 17KDa MW) ratio (**C,D**), but not at higher dose (Tic 10 μM). As shown in panels E-H, adenosine (ADE, 10 μM) *per se* does not enhance cell viability and proliferation and its addiction to culture medium counteracts the effects of Tic 1 μM. Adenosine is added in the presence of EHNA (10 μM). Levels of phospho-H3S10 and H3 are normalized on glyceraldehyde 3-phosphate dehydrogenase (GAPDH) levels and values of ratio are expressed as arbitrary units (a.u.). Representative images of cropped densitometric bands of proteins phospho-H3S10, H3 and GAPDH are showed in each panel. In Supplemental Fig. 6**:** the full-length blots/gels of panel C are presented as V, T1-_1_, T10-_1_ (**A**); the full-length blots/gels of panel G are presented as V2, A2, AT1-_2_, AT10-_2_ (**B**). All measurements are mean ± SD. *p < 0.05 vs. untreated condition (Vehicle: sterile phosphate buffer solution).

As shown in Fig. 2A,B, 72 h treatment with low-dose ticagrelor (1 μM) significantly increased viability and number of cultured hCPCs, but not at higher dose (10 μM). The elevated levels of phospho-H3S10/H3 in hCPCs treated with lower dose of drug defined threshold for proliferative effects of ticagrelor (Fig. 2C,D). Of note, ticagrelor did not alter H3 expression. This effect was not induced by long-term treatment of cells with exogenous adenosine per se, which instead counteracted the effects of low-dose ticagrelor and did not enhance the effects of high-dose ticagrelor on cell viability and proliferation (Fig. 2E–H).

### Reduced histone H3 phosphorylation inhibits ticagrelor-driven effects on hCPCs

**Figure 3:**
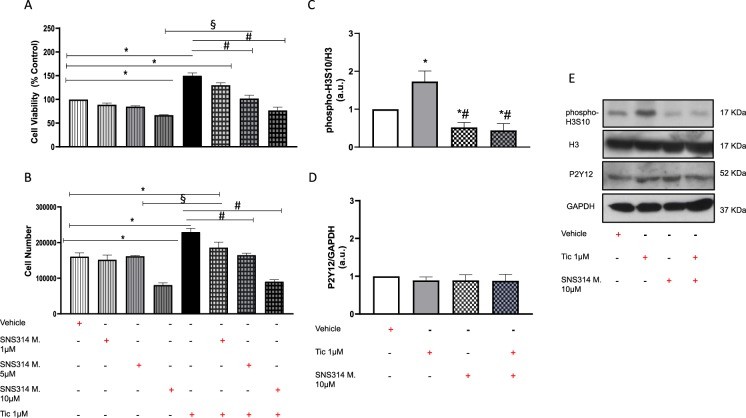
SNS314 mesylate (SNS314 M), an inhibitor phosphorylation of histone H3 at serine 10 (phospho-H3S10), reduces cell viability (**A**) and proliferation (**B**) in a dose dependent manner in hCPCs untreated and treated with ticagrelor (Tic 1 μM). Higher dose of SNS314 M (10 μM) counteracts ticagrelor (Tic 1 μM) effects (**A**,**B**). SNS314 M 10 μM counteract effects of Tic 1 μM on levels of phospho-H3S10/H3 ratio (**C**) without altering expression of P2Y_12_ (**D**; 52KDa MW) of hCPCs. Levels of phospho-H3S10, H3 and P2Y_12_ are normalized on glyceraldehyde 3-phosphate dehydrogenase (GAPDH) levels and expressed as arbitrary units (a.u.). Representative images of cropped densitometric bands of proteins phospho-H3S10, H3, P2Y_12_ and GAPDH are showed in panel E. The full-length blots/gels are presented in Supplemental Fig. 7 panel A. All measurements are mean ± SD. *p < 0.05 vs. untreated condition (Vehicle: sterile phosphate buffer solution); ^#^p < 0.05 vs. Tic 1 μM; ^§^p < 0.05 vs. SNS314 M (10 μM).

**Figure 4:**
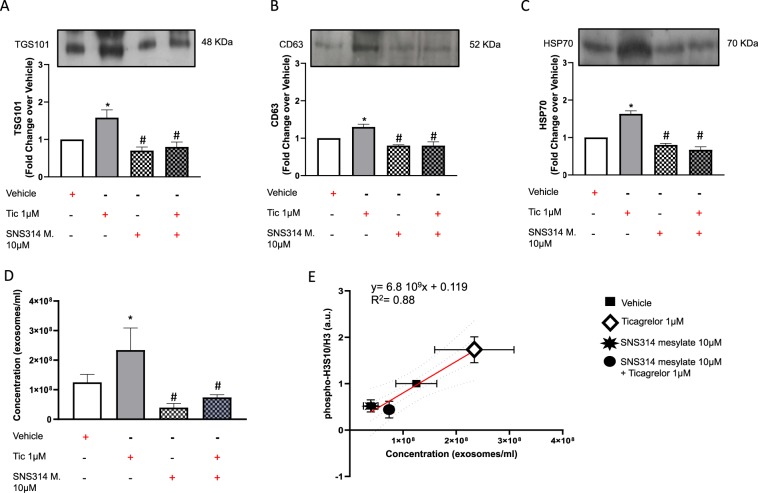
SNS314 mesylate (SNS314 M), an inhibitor phosphorylation of histone H3 at serine 10 (phospho-H3S10), counteracts the effects of ticagrelor (Tic) 1uM on levels of TSG101 + (**A**; 48KDa MW), CD63 + (**B**; 52KDa MW) and heat shock protein 70 (HSP70) + (**C**; 70KDa MW) exosomes. Representative images of cropped densitometric bands of proteins TSG101, CD63 and HSP70 are showed in each panel. The full-length blots/gels are presented in Supplemental Fig. 7 panel B. As confirmed in panel D, SNS314 M prevents rising of exosome concentration in culture medium induced by Tic 1 μM assessed by NanoSight technology. As shown in panel E, there is a positive correlation between changes in phospho-H3S10/H3 values and exosome concentration in each experimental condition. All measurements are mean ± SD. *p < 0.05 vs. untreated condition (Vehicle: sterile phosphate buffer solution); #p < 0.05 vs. Tic 1 μM.

As shown in Fig. 3, co-treatment of hCPCs with higher doses of SNS314 mesylate, an inhibitor of histone H3 phosphorylation, reverted the effects of ticagrelor to control level (vehicle) in terms of both cell viability (A) and number of cells (B) in a dose-dependent manner. In light of these results, selected dose of SNS314 mesylate (10 μM) prevented the ticagrelor-induced increase of phospho-H3S10/H3 ratio (C, E) without altering P2Y12 levels (D, E). As shown in Fig. 4, co-treatment with SNS314 mesylate prevented ticagrelor-induced medium enrichment in exosomes as assessed by the expression of TSG101 (A), CD63 (B) and HSP70 + (C). The relationship between extent of histone H3 phosphorylation and exosome levels was confirmed by NanoSight technology (Fig. 4D and Supplemental Fig. 4). Of importance, we found a positive relationship among phospho-H3S10/H3 values and exosome levels (Fig. 4E).

### Ticagrelor enhances anti-apoptotic effects of hCPCs-exosomes

**Figure 5:**
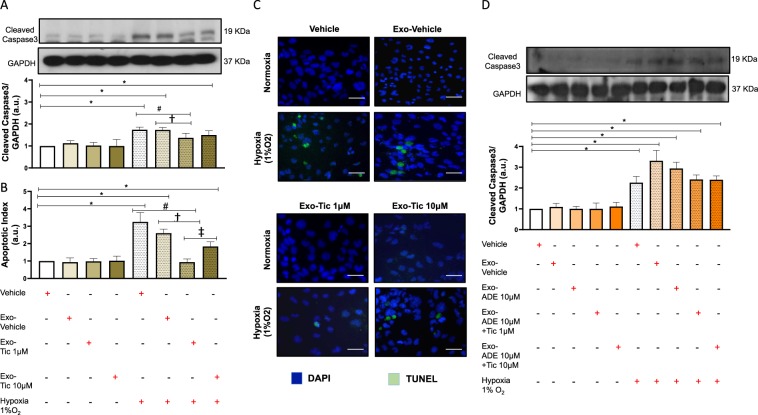
Long-term pre-treatment for 48 h of normoxic HL1 cardiomyocytes with exosomes (100 μg) derived from hCPCs treated with low-dose ticagrelor (Tic 1 μM) prevents rising of cleaved caspase 3 levels (**A**) and apoptosis index (**B**) during severe hypoxia (1% O_2_), but not exosomes derived from hCPCs treated with high-dose ticagrelor (Tic 10 μM). Representative images of TUNEL staining of HL1 cells in each experimental condition are showed in panel C. As shown in panels D, exosomes released from hCPCs treated in the presence of adenosine (ADE, 10 μM) and EHNA (10 *μ*M) do not prevents rising of cleaved caspase 3 levels in hypoxic cardiomyocytes. Representative images of full-length blots/gels of proteins cleaved caspase 3 and glyceraldehyde 3-phosphate dehydrogenase (GAPDH) are showed in panel A and D. Levels of cleaved caspase 3 are normalized on GAPDH levels and expressed as arbitrary units (a.u.) of cleaved caspase 3 (19 kDa, MW)/GAPDH (37 kDa, MW) ratio. All measurements are mean ± SD. *p < 0.05 vs. untreated condition (Vehicle: sterile phosphate buffer solution); ^#^p < 0.05 vs. hypoxia; ^†^p < 0.05 vs. Exo-Vehicle; ^‡^p < 0.05 vs. Exo-Tic 1 μM.

As shown in Fig. 5A, 48 h pre-treatment of HL1 cardiomyocytes with exosomes (100 μg) released by hCPCs primed with low-dose ticagrelor significantly prevented rising levels of cleaved caspase 3 due to chronic severe hypoxia. Anti-apoptotic effects of ticagrelor-induced exosomes were confirmed by TUNEL assay (Fig. 5B,C). Prevention of hypoxia-driven cardiomyocytes apoptosis was not observed after treatment with similar dose of exosomes released by hCPCs treated with vehicle or 10 μM ticagrelor (Fig. 5A,B) even in presence of adenosine (Fig. 5D). Interestingly, similar dose of exosomes released from hCPCs co-treated with 1 μM ticagrelor and adenosine were unable to prevent cardiomyocyte apoptosis due to sustained severe hypoxia (Fig. 5D).

### Ticagrelor-induced hCPCs exosomes activate ERK42/44 in cardiomyocytes

**Figure 6:**
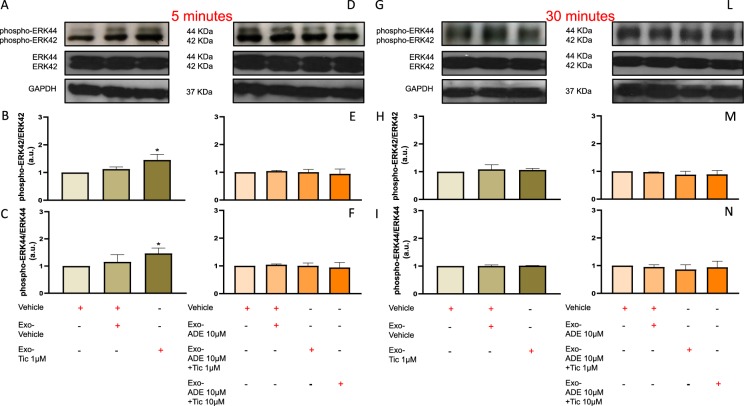
Long-term pre-treatment for 48 h of normoxic HL1 cardiomyocytes with exosomes (100 μg) derived from hCPCs treated with low-dose ticagrelor (Tic 1 μM) increase levels of phosphorylated ERK42/total ERK42 (**B**; 42KDa MW) and phosphorylated ERK44/ total ERK44 (**C**; 44KDa MW) ratios after 5 min. Representative images of cropped densitometric bands of phosphorylated and total ERK 42/44, and glyceraldehyde 3-phosphate dehydrogenase (GAPDH; 37KDa MW) are showed in panel A and D. All full-length blots/gels are presented in Supplemental Fig. 8 panel A. As shown in panels E,F, exosomes released from hCPCs treated in the presence of adenosine (ADE, 10 μM) and EHNA (10 μM) do not induce rising of intracellular phosphorylated ERK42/44 levels. Representative images of cropped densitometric bands of phosphorylated and total ERK 42/44, and GAPDH are showed in panel D. Intracellular levels of phosphorylation of ERK42/44 are normal in HL1 cells after 30 min of treatment with ticagrelor-induced exosomes in the absence (**H**,**I**) or presence (**L**,**M**) of adenosine (ADE 10 μM) + EHNA (10 μM). Representative images of cropped densitometric bands of phosphorylated and total ERK 42/44, and GAPDH are showed in panel G and L. All full-length blots/gels are presented in Supplemental Fig. 8 panel B. Levels of ERK42/44 are normalized on GAPDH levels and ratios are expressed as arbitrary units (a.u.). All measurements are mean ± SD. *p < 0.05 vs. untreated condition (Vehicle: sterile phosphate buffer solution).

As shown in Fig. 6, long-term exposure of HL1 cardiomyocytes with defined amount of exosomes (100 μg) released from hCPCs treated with 1 μM ticagrelor increased phosphorylation of ERK42 and 44 at 5 min (A-C), which decreased after 30 min (Figure H-I). The rapid phosphorylation of ERK42 and 44 was not induced by similar dose of exosomes released by hCPCs in other experimental conditions (Fig. 6A–C). Of note, similar dose of exosomes released from hCPCs treated with adenosine *per se* or co-treated with increasing doses of ticagrelor and adenosine did not increase phosphorylation of ERK42 and 44 at 5 (Fig. 6D–F) and 30 min (Fig. 6L–N).

### Ticagrelor-induced hCPCs exosomes attenuate the rise HIF1α levels in hypoxic cardiomyocytes

**Figure 7:**
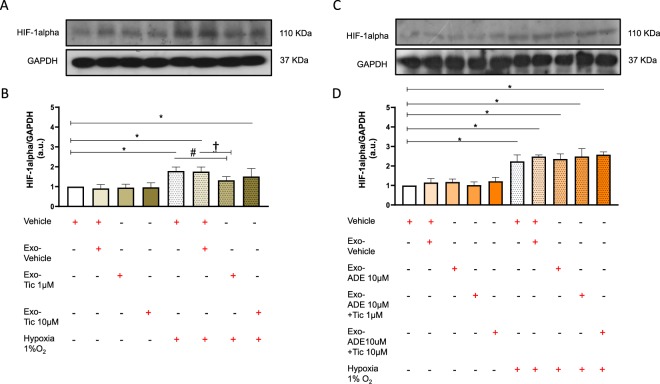
Long-term pre-treatment for 48 h of normoxic HL1 cardiomyocytes with exosomes (100 μg) derived from hCPCs treated with low-dose ticagrelor (Tic 1 μM) prevents rising of hypoxia inducible factor 1-alpha (HIF1-alpha; 110KDa MW) during severe hypoxia (1% O_2_), but not exosomes derived from hCPCs treated with high-dose ticagrelor (Tic 10 μM) (**B**). Representative images of full-length blots/gels of HIF1-alpha and glyceraldehyde 3-phosphate dehydrogenase (GAPDH; 37KDa MW) are showed in panel A. As shown in panels (**D**), exosomes released from hCPCs treated in the presence of adenosine (ADE, 10 μM) and EHNA (10 μM) do not reduce intracellular HIF1-alpha levels in hypoxic HL1. Representative images of full-length blots/gels of HIF1-alpha and GAPDH are showed in panel (C). Levels of HIF1-alpha are normalized on GAPDH levels and ratios are expressed as arbitrary units (a.u.). All measurements are mean ± SD. *p < 0.05 vs. untreated condition (Vehicle: sterile phosphate buffer solution); ^#^p < 0.05 vs. hypoxia; ^†^p < 0.05 vs. Exo-Vehicle.

As shown in Fig. 7, long-term exposure of HL1 cardiomyocytes with defined amount of exosomes (100 μg) released from hCPCs treated with low-dose ticagrelor attenuated the rise of intracellular HIF1α levels during severe hypoxia (A-B). Conversely, similar dose of exosomes released from hCPCs co-treated with 1 μM ticagrelor and adenosine were unable to attenuate HIF1α levels in hypoxic cardiomyocytes (Fig. 7C,D).

### P2Y12 antagonism by 2-oxo-clopidogrel does not recapitulate the ticagrelor-driven effects on release of anti-hypoxic exosomes from hCPCs

**Figure 8:**
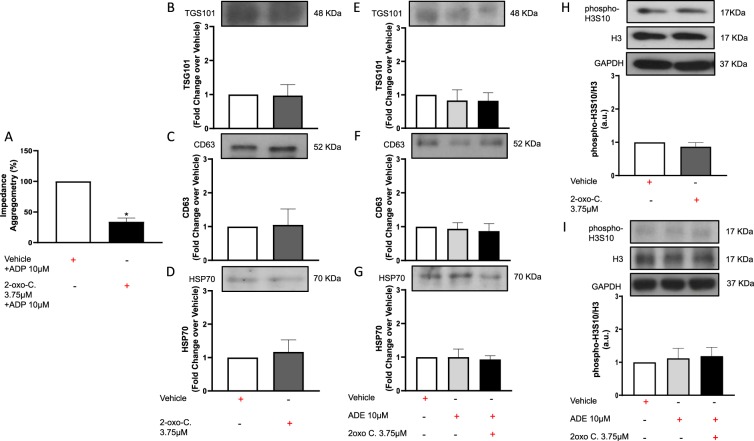
Long-term treatment for 72 h of human cardiac progenitor cells (hCPCs) with anti-aggregant dose (3.75 μM) of 2-oxo-clopidogrel (2-oxo-C) (**A**; in the presence of ADP 10 μM) does not recapitulate the ticagrelor-driven effects on release of TSG101 + (48KDa MW), CD63 + (52KDa MW) and heat shock protein (HSP)-70 + (70KDa MW) exosomes from hCPCs in the absence (**B–D**) or presence (**E–G**) of adenosine (ADE 10 μM) + EHNA (10 μM). Representative images of cropped densitometric bands of proteins TSG101, CD63, HSP70 are showed in each corresponding panel. The full-length blots/gels are presented in Supplemental Fig. 9 panel A. In addition, 2-oxo-C does not increase intracellular levels of phosphorylated histone H3 on Serine 10 (phospho-H3S10; 17KDa MW)/total histone H3 (H3; 17KDa MW) ratio in the absence (**H**) or presence (**I**) of adenosine (ADE 10 μM) + EHNA (10 μM). Representative images of cropped densitometric bands of proteins phospho-H3S10, H3 and glyceraldehyde 3-phosphate dehydrogenase (GAPDH) are showed in each corresponding panel. The full-length blots/gels are presented in Supplemental Fig. 9 panel B. Levels of each analyzed protein are normalized on GAPDH (37KDa MW) levels and ratios are expressed as arbitrary units (a.u.). All measurements are mean ± SD. *p < 0.05 vs. control condition.

**Figure 9:**
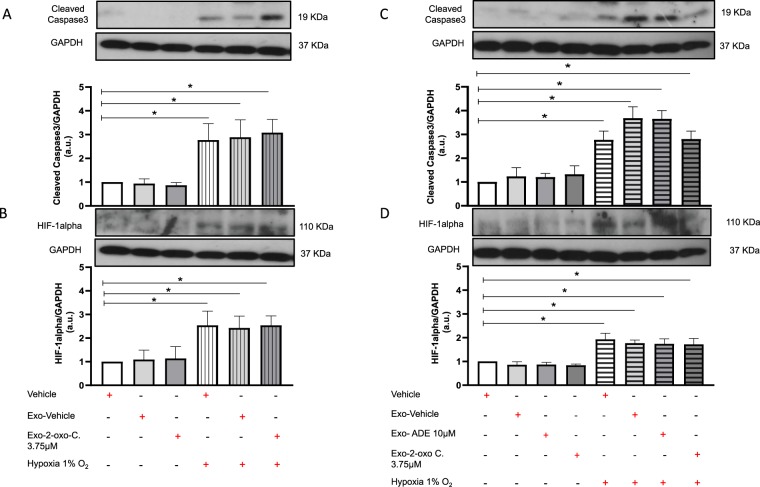
Long-term pre-treatment for 48 h of normoxic HL1 cardiomyocytes with exosomes (100 μg) derived from hCPCs treated with anti-aggregant dose (3.75 μM) of 2-oxo-clopidogrel (2-oxo-C) does not prevent rising of cleaved caspase 3 (19KDa MW) and hypoxia inducible factor 1-alpha (HIF1-alpha; 110KDa MW) during severe hypoxia (1% O_2_) in the absence (**A,B**) or presence (**C,D**) of adenosine (ADE 10 μM) + EHNA (10 μM). Representative images of cropped densitometric bands of proteins cleaved caspase 3, HIF1-alpha and glyceraldehyde 3-phosphate dehydrogenase (GAPDH) are showed in each corresponding panel. The full-length blots/gels of abovementioned proteins in absence or presence of adenosine (ADE 10 μM) + EHNA (10 μM) are presented in panel A and B of Supplemental Fig. 10 respectively. Levels of each analyzed protein are normalized on GAPDH (37KDa MW) levels and ratios are expressed as arbitrary units (a.u.). All measurements are mean ± SD. *p < 0.05 vs. untreated condition (Vehicle: sterile phosphate buffer solution).

As shown in Fig. 8, anti-aggregant dose of 2-oxo-clopidogrel, established in additional experiments (A), did not increase exosome levels as assessed by expression of TSG101 (B), CD63(C) and HSP70 (D). Moreover, additional exogenous adenosine did not enhance 2-oxo-clopidogrel effects on exosome release (Fig. 8E–G). Similarly, 2-oxo-clopidogrel with or without adenosine failed to increase phospho-H3S10/H3 ratio values (Fig. 8H–I). Finally, chronic pre-treatment of HL1 cardiomyocytes with exosomes released by CPCs treated with 2-oxo-clopidogrel did not prevent hypoxia-driven rising of cleaved caspase 3 (Fig. 9A,C) nor change HIF1α levels compared to untreated cells (Fig. 9B,D).

## Discussion

In the present study, we have demonstrated that long-term treatment of viable human cardiac-derived mesenchymal progenitor cells with 1 μmol ticagrelor increases the release of TGS101 + and CD63 + exosomes delivering anti-apoptotic HSP70 as consequence of increased number of viable hCPCs. Since the mean maximal total plasma concentrations after 4 weeks of ticagrelor treatment (60–90 mg bid) is 1–1.5 μmol/l^30^, our *in vitro* cell culture findings could support the development of novel clinically relevant pharmacological interventions for exosome-based cardioprotection, even suggesting a dose-dependent causal relationship with treatment.

Our data reveal an hitherto unexpected role of direct-acting P2Y_12_ receptor antagonist in regulating function of hCPCs, which are cells resident in human heart and potentially involved in protection of adult myocardium against ischemic injury through release of exosomes limiting cardiomyocytes apoptosis^31^. Although previous studies have demonstrated that chemical compounds may augment exosome release despite their toxicity^32^, the development of safe pharmacological approach to managing the local release of hCPCs-derived exosomes conferring pro-survival signals to hypoxic cardiomyocytes remains a desirable goal. Indeed, targeted therapies by means of compound that acts on a specific target receptor still represent an attractive perspective in cardioprotection. Since P2Y_12_ belongs to the family of G-protein coupled receptors, which signaling may trigger exosome release from adult mature cells^33^, we tested, for the first time, the regulatory role of ticagrelor, a cardioprotective inverse agonist of the P2Y_12_ receptor^34^, on exosome-mediated paracrine effects of explant-derived hCPCs. Like human hematopoietic stem/progenitor cells^27^ and vascular smooth muscle cells, we have demonstrated that P2Y_12_ receptors are also expressed in hCPCs.

Significantly, long-term treatment of hCPCs with clinically relevant dose of ticagrelor increases levels of TGS101 + , CD63 + and HSP70 + exosomes in culture medium. Of importance, this effect is not observed during treatment with higher dose of ticagrelor. Since exosome levels in culture medium depend on cell viability and densities^35^, we assessed dose-dependent effects of ticagrelor on viability and proliferation of cultured normoxic hCPCs. Interestingly, long-term treatment for 72 h with only 1μmol ticagrelor increases the number of viable hCPCs and the magnitude of phospho-H3S10/H3 ratio, an established hallmark of progenitor cells proliferation^36^. Like exosome levels, ten-fold higher dose of ticagrelor does not affect cell viability and proliferation. Our data exclude dose-dependent toxicity of the drug and reveal a positive relationship between extracellular exosome levels and magnitude of mitotic activity of human explant-derived cardiac stromal cells during ticagrelor treatment *in vitro*. These findings are well supported by previous clinical study showing an increased number of circulating endothelial progenitor cells in patients with acute coronary syndrome treated with ticagrelor^37^. In order to assess underlying mechanisms, we first investigated the role played by adenosine, an established mediator of ticagrelor effects.

Although it is known that some exosomes may increase proliferation of non-tumoral cells through autocrine vicious cycle^38^, it is conceivable that rising of exosome levels related to higher cell proliferation rate might be mediated by ticagrelor-induced adenosine. Although cultured normoxic cells at resting conditions release adenosine in the nanomolar range^39^, P2Y_12_ antagonism by ticagrelor may increase extracellular adenosine levels due to both dose-dependent ATP release^40^ and inhibition of the equilibrative nucleoside transporter 1 (ENT1)^41^, an adenosine transporter even expressed in different types of progenitor cells^42,43^. Therefore, extracellular adenosine amount following treatment with clinically relevant dose of ticagrelor might be enough to induce hCPCs proliferation through activation of high-affinity adenosine receptors, such as A_1_ and A_2A_. Indeed, activation of A_1_ and A_2A_ adenosine receptor promotes neural progenitor cells proliferation^44^. Conversely, exposure of cells to ten-fold higher dose of ticagrelor further increases extracellular adenosine levels^19^ and activates A_2B_^45^, a low-affinity adenosine receptor even expressed by cardiac mesenchymal stem-like cells^46^ that arrest cell cycle^46^. In order to highlight the dose-dependent role of adenosine as mediator of ticagrelor effects, we have also performed further experiments by adding exogenous adenosine. Indeed, the effects of low-dose ticagrelor on exosome release and cell proliferation are counteracted by co-treatment with higher dose of exogenous adenosine and EHNA, a potent inhibitor of extracellular adenosine deaminase^47^ that prevents adenosine degradation. Of note, the evidence that high concentration of adenosine *per se* does not prompt rising of exosome levels in the culture medium and cell proliferation further support the regulatory role played by ticagrelor-induced adenosine at lower rather than higher dose.

Lower extracellular adenosine levels, through its A_2A_ receptor, can activate tyrosine kinases and influence several signaling pathways involved in cell proliferation including the PI3-kinase/Akt pathway and the ERK/MAPK pathways^48^, whose activation induces histone H3 phosphorylation at serine 10^49^. However, similar activation of the MAPK signaling pathway can also increase the number of exosomes released per cell^50^. To investigate the downstream pathways involved in ENT1-dependent activation of A_2A_ receptor, we have pre-treated explant-derived hCPCs with nontoxic dose of SNS314 mesylate, an inhibitor of histone H3 phosphorylation at serine 10 through inhibition of Aurora kinases that are downstream MAPK^51^. Interestingly, the subsequent treatment of these cells with clinically relevant dose of ticagrelor fails to increase proliferation rate and exosome concentration is similar to control cells. Therefore, we suggest that A_2A_-dependent effects of ticagrelor on exosome release are mainly due to regulation of histone H3 phosphorylation at serine 10.

Given that lack of ENT1 is cardioprotective^52^, it may play a key role in modulating A_2A_-dependent effects of ticagrelor on mitotic activity of hCPCs and exosome release. For this purpose, we have performed additional experiments by treating hCPCs with anti-aggregant nontoxic dose of 2-oxo-clopidogrel, an irreversible P2Y_12_ antagonist that does not activate ENT1^41^. Interestingly, 2-oxo-clopidogrel does not increase levels of extracellular exosome levels and histone H3 phosphorylation at serine 10, even in the presence of exogenous adenosine and EHNA. Thus, our data suggest that inhibition of ENT1 is required to mediate ticagrelor effects.

It is well established that hCPCs-derived exosomes exert anti-apoptotic effects *in vitro* on cardiomyocytes undergoing starvation^21^ or treated with pro-apoptotic staurosporine^23^, as well in rodent model of myocardial infarction^21–23,52^. However, their anti-hypoxic effects have never been addressed. In fact, the intensity and severity of hypoxic stress promote cell injury through different regulatory mechanisms compared to those induced by glucose or serum deprivation^53^. Given the emerging interest in testing the effect of conventional cardiovascular drugs on the cardioprotective potential of exosomes derived from stem/progenitor cells^54^, we have assessed the role of ticagrelor-induced hCPCs-derived exosomes in increasing resistance of cardiomyocytes to severe hypoxia. We found, for the first time, that exosomes released from auricle-derived hCPCs treated with low-dose ticagrelor attenuates hypoxia-induced caspase-3 activation in HL1 cardiomyocytes. Since anti-apoptotic effects of hCPCs-derived exosomes are dose-dependent^21^, similar dose of hCPCs-derived exosomes released in other experimental conditions is not effective in preventing hypoxia-induced apoptosis at least in our *in vitro* setting.

The present results highlight the new role of ticagrelor in enhancing anti-hypoxic properties of hCPCs-derived exosomes. Greater anti-hypoxic effects of exosomes induced by low-dose ticagrelor might be related to HSP70, a well-known inducible protein that protects against hypoxia-induced apoptosis^55^. Although ticagrelor does not increase HSP70 expression of hCPCs, it is plausible that ticagrelor may expose cardiomyocytes to extracellular milieu rich of exosomal HSP70 released from resident hCPCs.

Exosomal HSP70 is essential to protect adult cardiomyocytes through rapid ERK42/44 phosphorylation^56^ and its activity depends on the microenvironment surrounding cell source. Indeed, rodent and human exosomes released under hyperglycemic conditions show either lower HSP70 levels or HSP70 inactivated by non-enzymatic glycosylation that are unable to prevent apoptosis of cardiomyocytes after hypoxia and re-oxygenation^57^. In our *in vitro* model, ERK42/44 phosphorylation is rapidly increased after 5 min of exposure of HL1 to exosomes derived from hCPCs treated with clinically relevant dose of ticagrelor. Conversely, there was no significant increase in ERK42/44 phosphorylation after treatment with similar dose of exosomes released in other experimental conditions. In order to further support the exosome-based anti-hypoxic effect, we measured cardiomyocyte HIF1α levels, a transcription factor activated by low oxygen concentrations, which expression is downregulated when hypoxia-induced oxidative stress and cardiomyocyte apoptosis are attenuated by other agents^58^. In our experimental setting, HIF1α levels were significantly lower in viable HL1 cardiomyocytes pre-treated with exosomes, released after treatment of hCPCs with low-dose ticagrelor, and then exposed to severe hypoxia. Our results are in line with previous study showing that intracellular enrichment of HSP70 inhibits the expression of HIF1α by protecting hypoxic cells^59^. Indeed, hCPCs-derived exosomes are effectively internalized by cardiomyocytes^21–23,26^.

### Limitations of the study

In our study we did not performed analytical approach for analyzing the cargo of single hCPC-derived exosome in each experimental condition in order to evaluate the effects of clinically relevant dose of ticagrelor on exosomal content. Although pro-survival effects of HSP70 are supported by the present results and by others^56^, we cannot exclude that other proteins may synergize the anti-hypoxic effects of ticagrelor-induced exosomes. Despite our limited understanding of cardiac-derived mesenchymal progenitor cells function *in vivo* in terms of cardiac regeneration, these cells reside in human heart^23,26^ and may be a potential target of ticagrelor in mediating exosome-based cardiomyocyte protection. However, further experiments should be performed in an *in vivo* setting in order to confirm our *in vitro* results supporting wide evidence of ticagrelor-induced cardioprotection.

### Conclusions

To the best of our knowledge, this is the first evidence demonstrating that clinically relevant dose of ticagrelor acts on human cardiac-derived mesenchymal progenitor cells safely increasing the release of anti-hypoxic exosomes. This effect is achieved through the simultaneous rising of proliferation rate of viable hCPCs, which requires ENT1 activation and is counteracted by higher dose of adenosine. Ticagrelor-induced exosomes early activate pro-survival protein kinase signaling pathways including ERK42/44 by protecting hypoxic cardiomyocytes. Our results assume clinical significance in the development of new noninvasive pharmacological approach to enhance endogenous exosome-based anti-hypoxic response and protect heart at risk for developing myocardial ischemic injury.

## Methods

### Chemicals

Ticagrelor (Tic) was kindly provided by AstraZeneca (Mölndal, Sweden). Adenosine (ADE), a purine nucleoside mainly derived from the metabolism of ADP or adenosine triphosphate after myocardial damage^60^, and erythro-9- (2-hydroxy-3-nonyl) adenine (EHNA) hydrochloride, a potent adenosine deaminase inhibitor^47^, were purchased from Sigma-Aldrich Chemical Co (Missouri, USA). 2-oxo-clopidogrel, a key intermediate metabolite from which the active metabolite of clopidogrel is rapidly formed via oxidation^61^, was purchased from Santa Cruz Biotechnology (CA, USA). SNS314 mesylate, an ATP-competitive and selective pan-Aurora kinase inhibitor leading to inhibition phosphorylation of histone H3 at serine 10^51^, was purchased from Cayman Chemical (Michigan, USA).

### Cell Lines

hCPCs were isolated from the right atrial appendage of adult non-diabetic patients (n = 5; M/F = 3/2; mean age, 71.2 ± 7.82 years) with normal LV ejection fraction (56.6 ± 9.5%) undergoing open-heart surgery for coronary artery bypass grafting alone (n = 3) or combined to heart valve surgery (n = 2) as previously described by us^21,23^. Protocols used in this study to isolate hCPCs were approved by Ethical Committee for Clinical Research of Ticino Canton, Switzerland (Rif. CE 2923) and performed according to the Declaration of Helsinki. All patients gave written informed consent to the collection of atrial specimen. hCPCs were growth in Iscove’s Modified Dulbecco’s Medium (IMDM, Sigma-Aldrich Chemical Co, Missouri, USA) supplemented with 20% exosome-depleted fetal bovine serum (FBS), L-glutamine (Sigma-Aldrich Chemical Co, Missouri, USA) and antibiotics.

Murine HL-1 cardiomyocytes (a kind gift of Professor W. Claycomb, Department of Biochemistry and Molecular Biology, LSUHSC, New Orleans, LA, USA), a cell line used to study cardiomyocytes structure and function^62^ and exosomes effects^21,23^, were cultured in Claycomb Medium (Sigma-Aldrich Chemical Co, MO, USA) supplemented with 10% exosomes-depleted FBS, antibiotics, L-glutamine, and 100 μM norepinephrine (Sigma-Aldrich Chemical Co, MO, USA). All assays were conducted using low cell passage cells (2–5 passages).

### Flow cytometry

Surface markers expressed on explant-derived hCPCs were analyzed by flow cytometry. Phycoerythrin (PE)-conjugated antibodies against surface markers expressed on cardiac mesenchymal stromal cells (CD90, CD105, CD73, CD45, CD117; all from Beckman Coulter) were used. Beads incubated with an antibody in the absence of cells served as a control. Analyses were performed on a MACS-Quant flow cytometer (Miltenyi Biotec).

### Experimental protocol

hCPCs, seeded at density of 5 × 10^5^, were treated for 72 h with sterile phosphate buffer solution (PBS), non-cytotoxic increasing doses of ticagrelor (1 or 10 μM) or 2-oxo-clopidogrel (3.75 μM) when they reached about 80% confluence. Each chemical compound was added to IMDM medium supplemented with exosome-depleted FBS, L-glutamine and antibiotics at 37 °C in humidified air with 5% CO_2_. All experiments were performed with or without exogenous adenosine (10 μM) + EHNA (10 μM) or SNS314 mesylate (10 μM). At the end of each experimental treatment, culture medium was collected and exosomes were isolated as previously described by us^21,23^. Exosome pellets were suspended in PBS, aliquoted and stored at −80 °C until further use. Exosome concentrations were assessed using Western blot assay to measure TGS101, CD63 and HSP70 protein levels, well-established exosomes markers^63^, and by NanoSight technology (NanoSight LM10, Malvern Instrument, Amesbury, UK) as previously described by us^21,23^.

### MTT cell viability assay

After each pharmacological treatment, the cell viability was assessed using the 3-(4,5-dimethylthiazolyl-2)-2,5-diphenyltetrazolium bromide (MTT) reduction assay (Sigma-Aldrich Chemical Co, MO, USA) according to the manufacturer’s instructions. All measurements were performed in triplicate.

### Trypan blue assay and hemocytometer cell count

Trypan blue dye exclusion test by staining cells with 0.2% Trypan blue solution (Sigma-Aldrich Chemical Co, MO, USA) was performed to reveal necrotic cells after each experimental condition according to the manufacturer’s instructions. Moreover, unstained viable cells were manually counted using a hemocytometer. Counts were performed by triplicate under a 10X objective according to standard methodology^64^. All measurements were performed in triplicate.

### Apoptosis assay of HL-1 cardiomyocytes

HL-1 cardiomyocytes were seeded at a density of 6 × 10^4^/well in six-well culture dishes and allowed to reach ~80% confluence. Cells were pre-treated for 48 h with hCPC-exosomes (100 μg) isolated from culture medium as previously reported by us^21^. HL-1 cells treated with different hCPC-exosomes were randomly exposed for 24 h to severe hypoxia (1–2% O2) using a hypoxia incubator chamber (STEMCELL Technologies Inc., Canada), a well-established model of cardiomyocytes damage following myocardial infarction^27^, or to normoxia (21%O2) as control condition. Detection of TUNEL staining by fluorescence microscopy (*in situ* cell death detection kit, Roche Diagnostic Corporation, Indianapolis, IN, USA)^65^ and measurement of cleaved caspase-3 levels by Western blot assay^66^ were performed to evaluate the magnitude of hypoxia-induced apoptosis of HL-1 cardiomyocytes. All measurements were performed in triplicate.

### Western blot assay

hCPCs-exosomes, CPCs and HL-1 pellets were homogenized at 4 °C in RIPA buffer containing protease and phosphatase inhibitors (Pierce, Rockford, USA). Homogenates were centrifuged at 12,000 g for 15 minutes at 4 °C to remove nuclei and cell debris. Protein concentration in supernatant was determined using BCA protein assay kit (Pierce, Rockford, USA). Equal amounts of protein were fractionated by 8–15% SDS polyacrylamide gel and transferred to nitrocellulose membrane (Bio-Rad Laboratories Inc., CA, USA). Equal loading was controlled by Ponceau staining. Membranes were blocked with 5% nonfat dried milk in TBS/Tween20 (0,01%) at room temperature for 1 hour, and then probed with primary antibodies at a predetermined concentration at 4 °C overnight. Primary antibodies were used to detect: (1) human CD63 (polyclonal antibody, 1:1000; #SAB4301607, Sigma-Aldrich Chemical Co, MO, USA) and human TGS101 (polyclonal antibody, 1:1000; #T5701, Sigma-Aldrich Chemical Co, MO, USA) which are established exosomal markers^63^; (2) human heat shock protein 70 (HSP70) (polyclonal antibody, 1:1000; #ADI-SPA-812F, Enzo Life Sciences, NYC, USA), an antiapoptotic protein even used as exosomal marker^56^; (3) human P2Y_12_ on surface of CPCs (polyclonal antibody, 1:1000; #SC27152, Santa Cruz Biotechnology Inc., USA); (4) human phosphorylated Histone H3 on Serine 10 (phospho-H3S10) (polyclonal antibody, 1:1000; #9701 S, Cell Signaling, Danvers, MA) and human histone H3 (H3) (monoclonal antibody, 1:1000; #4499 S, Cell Signaling, Danvers, MA), which ratio is an established hallmark of cell proliferation^36^; (5) murine cleaved caspase-3 (polyclonal antibody, 1:1000; #SC7148, Santa Cruz Biotechnology Inc., USA), a marker of cell apoptosis^66^; (6) murine phospho-ERK42/44 (polyclonal antibody, 1:1000; #SC23759, Santa Cruz Biotechnology Inc., USA) and murine ERK42/44 (polyclonal antibody, 1:2000; #SC94, Santa Cruz Biotechnology Inc., USA) in HL-1 cells, which ratio is an established hallmark of exosome-mediated pro-survival pathway^23,56^; (7) murine hypoxia inducible factor 1-alpha (HIF1α; monoclonal antibody, 1:1000; #ab179483, Abcam, UK), a transcription factor activated by low oxygen concentrations^67^; (8) glyceraldehyde 3-phosphate dehydrogenase (GAPDH; monoclonal antibody, 1:3000; #G8795, Sigma-Aldrich Chemical Co, MO, USA) as loading control for protein normalization. After incubation with the above-mentioned primary antibodies, and rinsing with TBS/Tween20 (0.01%) 3 times for 10 min, membranes were incubated with appropriate horseradish peroxidase-conjugated (HRP-conjugated) anti-rabbit (#A0545), anti-goat (#A5420) or anti-mouse (#A9044) secondary antibodies (Sigma-Aldrich Chemical Co, MO, USA) for 1 hour at room temperature. After incubation with the specific secondary antibodies, membranes were washed 3 times for 10 minutes with TBS/Tween20 (0.01%). Specific protein bands were detected using ECL Plus Western Blotting Detection System (Pierce, Rockford, USA). Densitometry analysis of protein bands was performed using ImageJ software (National Institute of Health, USA). All measurements were performed in triplicate.

### Platelet aggregometry assay

Impedance aggregometry was performed with an optical aggregation system (CHRONO-LOG series 590, Havertown, PA), equipped with an infrared light beam and the AGGRO/LINK software^68^. Human whole blood sample (500 μl) was diluted 50:50 in 0.9% saline solution and heated for 5 min at 37 °C while stirring. After baseline measurement, platelet aggregation was evaluated after adding ADP (10 μM) in presence or absence of 2-oxo-clopidogrel (3.75 μM). The increase of impedance was recorded for 10 min. All measurements were performed in triplicate.

### Statistical analysis

All results are presented as mean ± standard deviation (SD). Statistical comparisons were made by analysis of variance (ANOVA) and Bonferroni test was used as the post-hoc test. Value p < 0.05 was considered statistically significant using GraphPad PRISM software (GraphPad Software Inc., San Diego, California).

## Supplementary information

Supplementary Material.
