# Supplementary material for: Ticagrelor Enhances Release of Anti-Hypoxic Cardiac Progenitor Cell-Derived Exosomes Through Increasing Cell Proliferation In Vitro
Source: Sci Rep. 2020 Feb 12;10:2494. doi: 10.1038/s41598-020-59225-7 (PMC7016113; doi:10.1038/s41598-020-59225-7)
Supplement: Supplementary file 1 — Supplementary Material. [file 41598_2020_59225_MOESM1_ESM.pdf]

Supplemental Material

Ticagrelor Enhances Release of Anti-Hypoxic Cardiac Progenitor Cell-Derived Exosomes Through Increasing Cell Proliferation In Vitro

Valentina Casieri MSc[1], Marco Matteucci PhD[1], Emilio M. Pasanisi MD[2], Angela Papa Msc[2], Lucio Barile PhD[3,4], Regina Fritsche-Danielson PhD[5], Vincenzo Lionetti MD, PhD[1,2*]

[1]Institute of Life Sciences, Scuola Superiore Sant'Anna, Pisa, Italy; [2]Fondazione Toscana G. Monasterio, Pisa, Italy; [3]Laboratory for Cardiovascular Theranostics, Cardiocentro Ticino Foundation, 6900 Lugano, Switzerland; [4]Faculty of Biomedical Sciences, Università Svizzera Italiana, 6900 Lugano, Switzerland; [5]Research and early clinical development, Cardiovascular, Renal and Metabolism, Biopharmaceuticals R&D, AstraZeneca, Gothenburg, Sweden.

Supplemental figure legends

Supplemental Figure 1. Surface marker expression profile in cardiac progenitor cells isolated form human auricle analysed by flow cytometry. Cardiac-derived mesenchymal progenitor cells express mesenchymal/stromal markers (CD90, CD73, CD105), but not the common leukocyte antigen CD45. CD117 is almost absent.

Supplemental Figure 2. Western blot representative image of $P2Y_{12}$ (52KDa MW) expression in human cardiac progenitor cells (hCPCs) and human vascular smooth muscle cells (VSMCs) in presence of normal protein loading as shown by glyceraldehyde 3-phosphate dehydrogenase (GAPDH; 37KDa MW) bands.

Supplemental Figure 3. Long-term treatment for 72h of human cardiac progenitor cells (hCPCs) with increasing dose of ticagrelor (Tic) does not alter intracellular expression of heat shock protein (HSP)-70. Representative images of cropped densitometric bands of proteins HSP70 are showed in panel A and full-length Western blots are showed in panel B. Levels of HSP70 are normalized on glyceraldehyde 3-phosphate dehydrogenase (GAPDH) levels and values are expressed as arbitrary units (a.u.). The full-length blots/gels of HSP70 and GAPDH proteins are shown in panel B. All measurements are mean ± SD.

Supplemental Figure 4. Representative images of dynamic light scatter analyses of particle size and concentrations in each experimental condition by NanoSight Technology.

Supplemental Figure 5. Western blots assay of hCPCs-derived exosomes. Panel A and B show the full-length Western blots corresponding to cropped blots in the main text.

Supplemental Figure 6. Western blots assay of explant-derived hCPCs. It is shown the full-length Western blots corresponding to cropped blots in the main text.

Supplemental Figure 7. Western blots assay of explant-derived hCPCs (A) and hCPCs-derived exosomes (B). Panel A and B show the full-length Western blots corresponding to cropped blots in the main text.

Supplemental Figure 8. Western blots assay of HL1 cardiomyocytes. Panel A and B show the full-length Western blots corresponding to cropped blots in the main text.

Supplemental Figure 9. Western blots assay of explant-derived hCPCs (A) and hCPCs-derived exosomes (B). Panel A and B show the full-length Western blots corresponding to cropped blots in the main text. Panel A and B show the full-length Western blots corresponding to cropped blots in the main text.

Supplemental Figure 10. Western blots assay of HL1 cardiomyocytes. Panel A and B show the full-length Western blots corresponding to cropped blots in the main text.

Supplemental Figure 1.

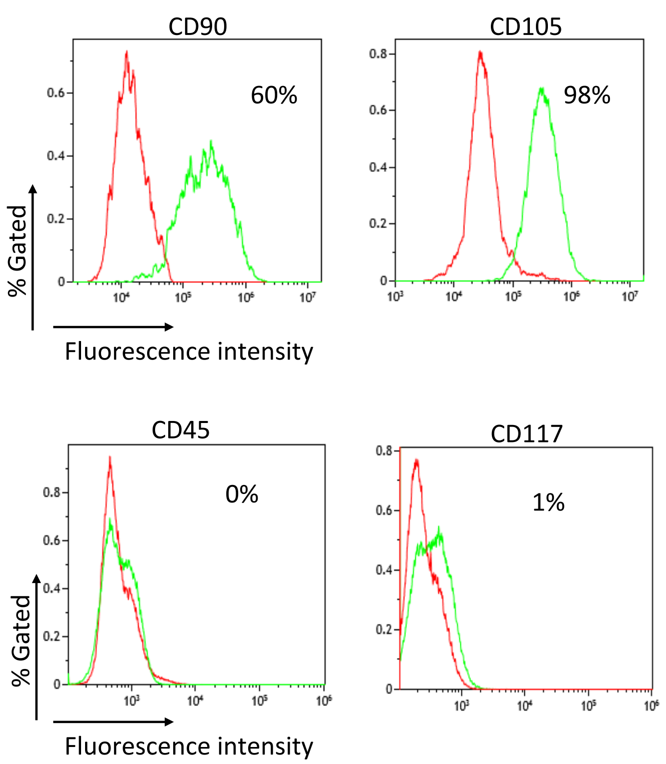

Supplemental Figure 2

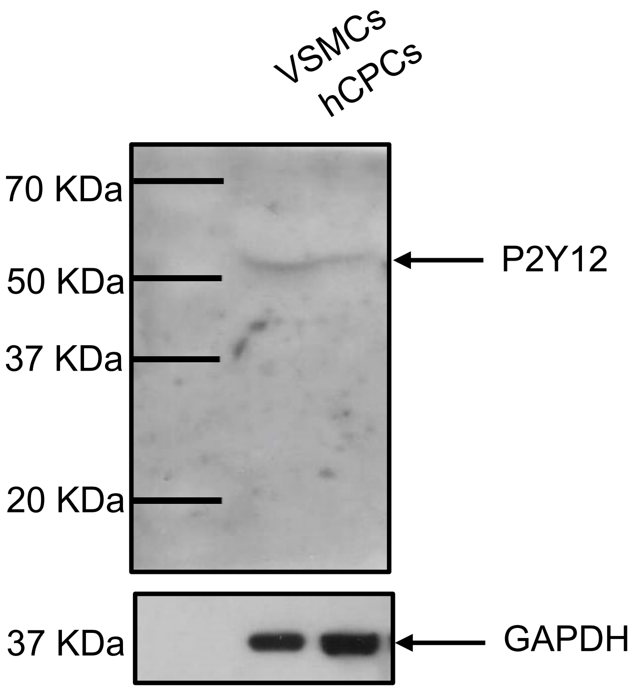

Supplemental Figure 3.

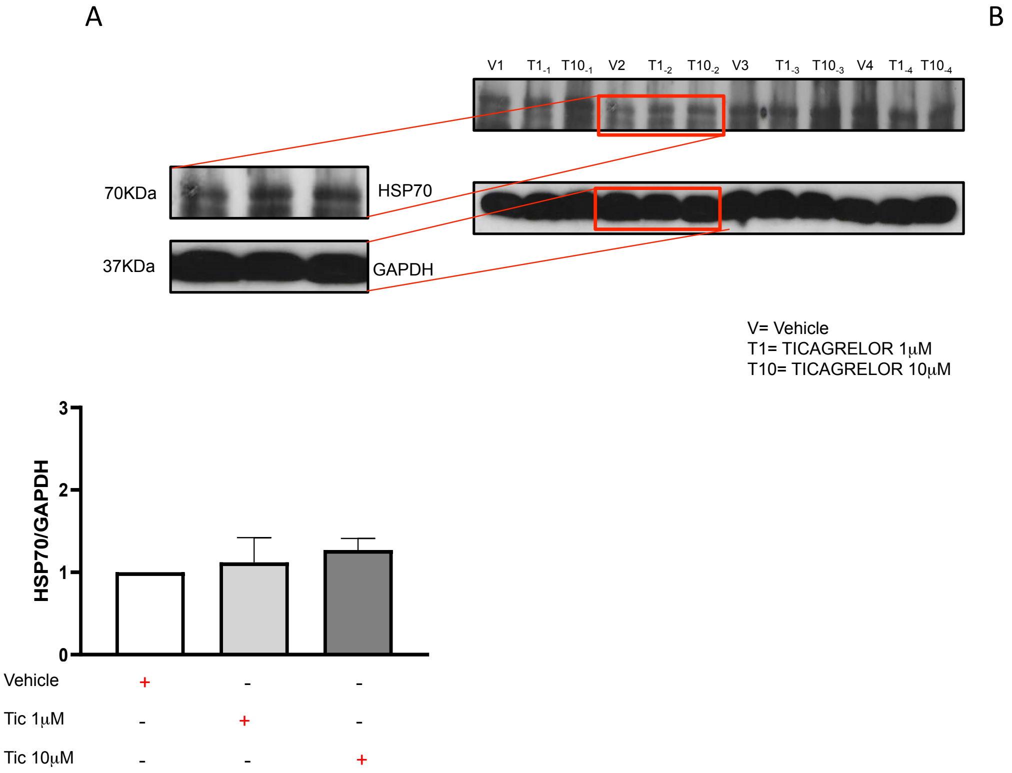

Supplemental Figure 4.

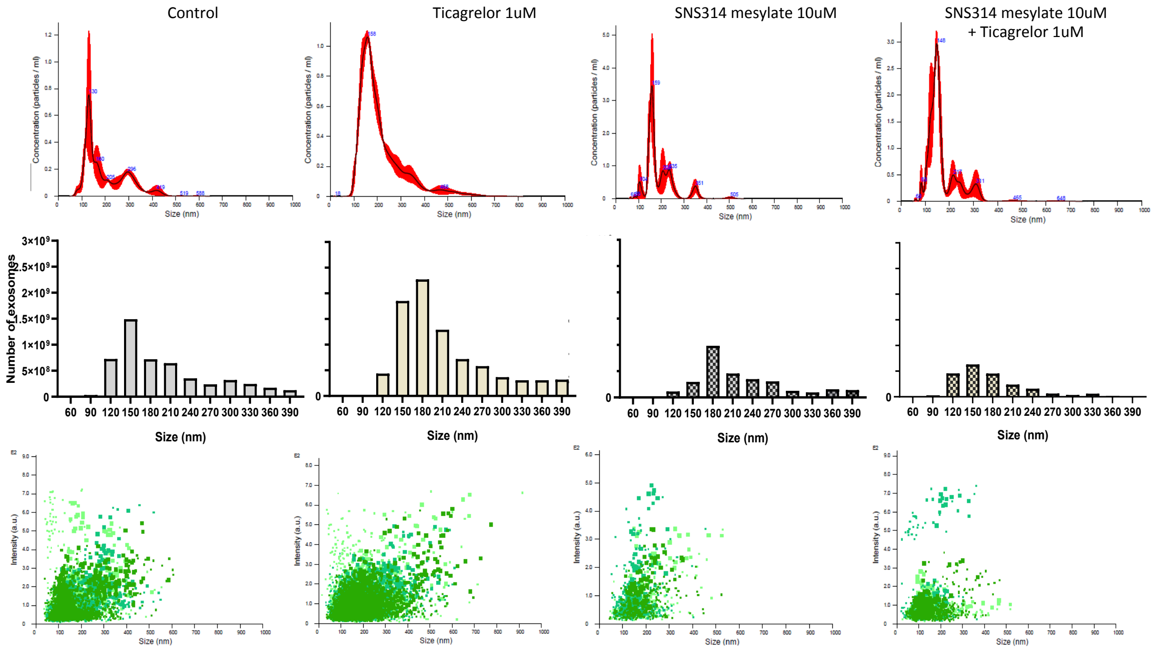

Supplemental Figure 5.

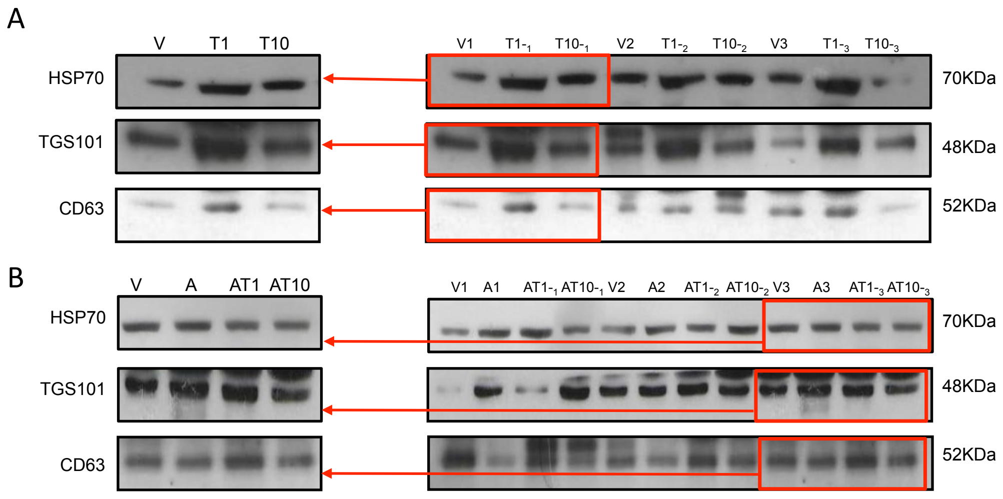

V= Vehicle
T1= TICAGRELOR 1μM
T10= TICAGRELOR 10μM
A= ADENOSINE 10μM
AT1= ADENOSINE 10μM+Ticagrelor 1μM
AT10 = ADENOSINE 10μM+ Ticagrelor 10μM

Supplemental Figure 6.

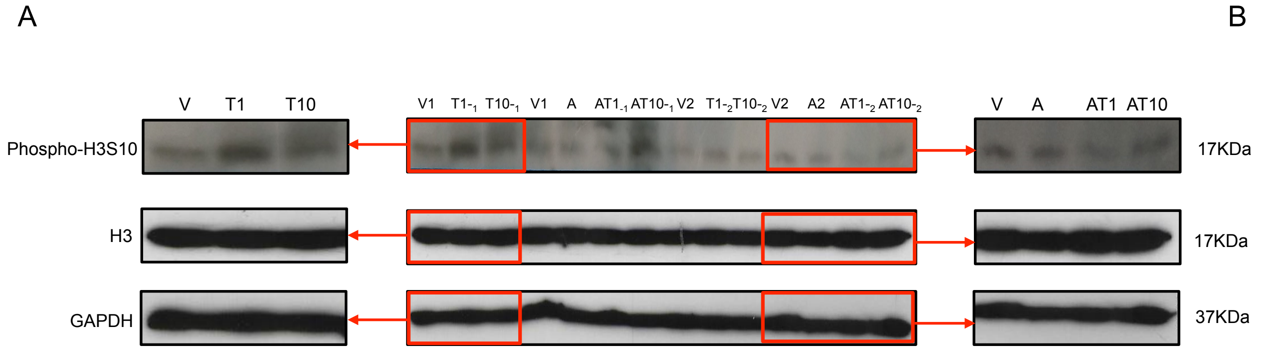

V= Vehicle
T1= TICAGRELOR 1μM
T10= TICAGRELOR 10μM
A= ADENOSINE 10μM
AT1= ADENOSINE 10μM+Ticagrelor 1μM
AT10 = ADENOSINE 10μM+ Ticagrelor 10μM

Supplemental Figure 7

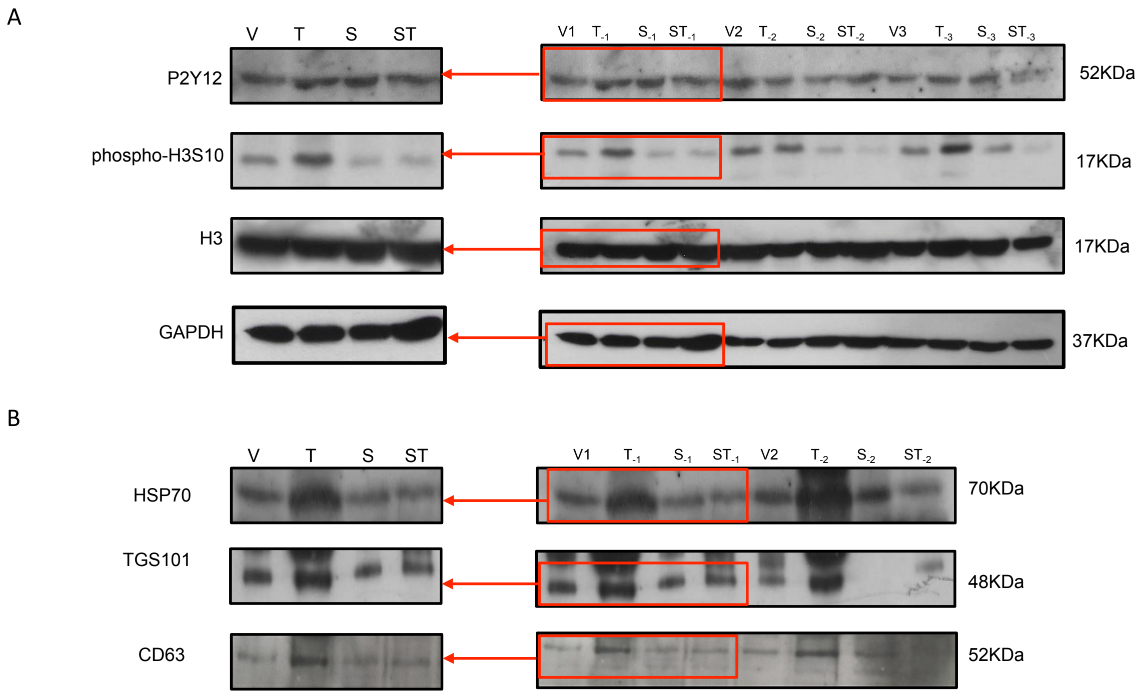

V= Vehicle
T= TICAGRELOR 1μM
S= SNS314-mesylate 10μM
ST= SNS314-mesylate 10μM+ TICAGRELOR 1μM

Supplemental Figure 8.

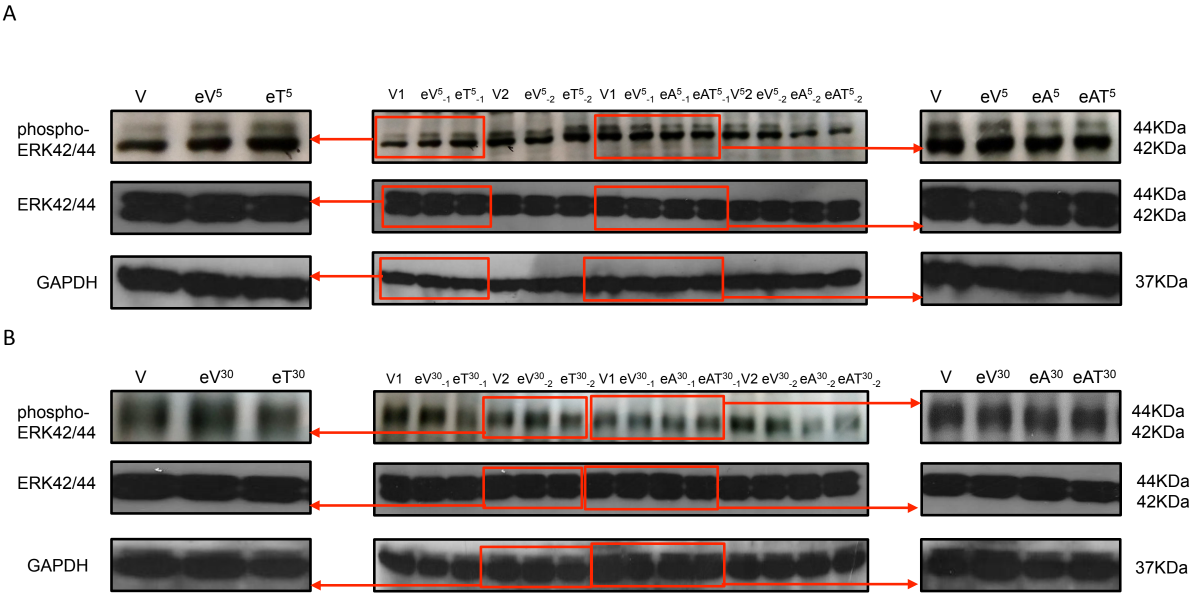

V= Vehicle
eV^{5}= exo-VEHICLE 5 MINUTES
eT^{5} = exo-TICAGRELOR 1μM 5 MINUTES

V= Vehicle
eV^{30}= exo-VEHICLE 30 MINUTES
eT^{30} = exo-TICAGRELOR 1μM 30 MINUTES

V= Vehicle
eV^{5}= exo-VEHICLE 5 MINUTES
eA^{5}= exo-ADENOSINE 10μM 5 MINUTES
eAT^{5} = exo-ADENOSINE 10μM+TICAGRELOR 1μM 5 MINUTES

V= Vehicle
eV^{30}= exo-VEHICLE 30 MINUTES
eA^{30}= exo-ADENOSINE 10μM 30 MINUTES
eAT^{30}= exo-ADENOSINE 10μM+TICAGRELOR 1μM 30 MINUTES

Supplemental Figure 9

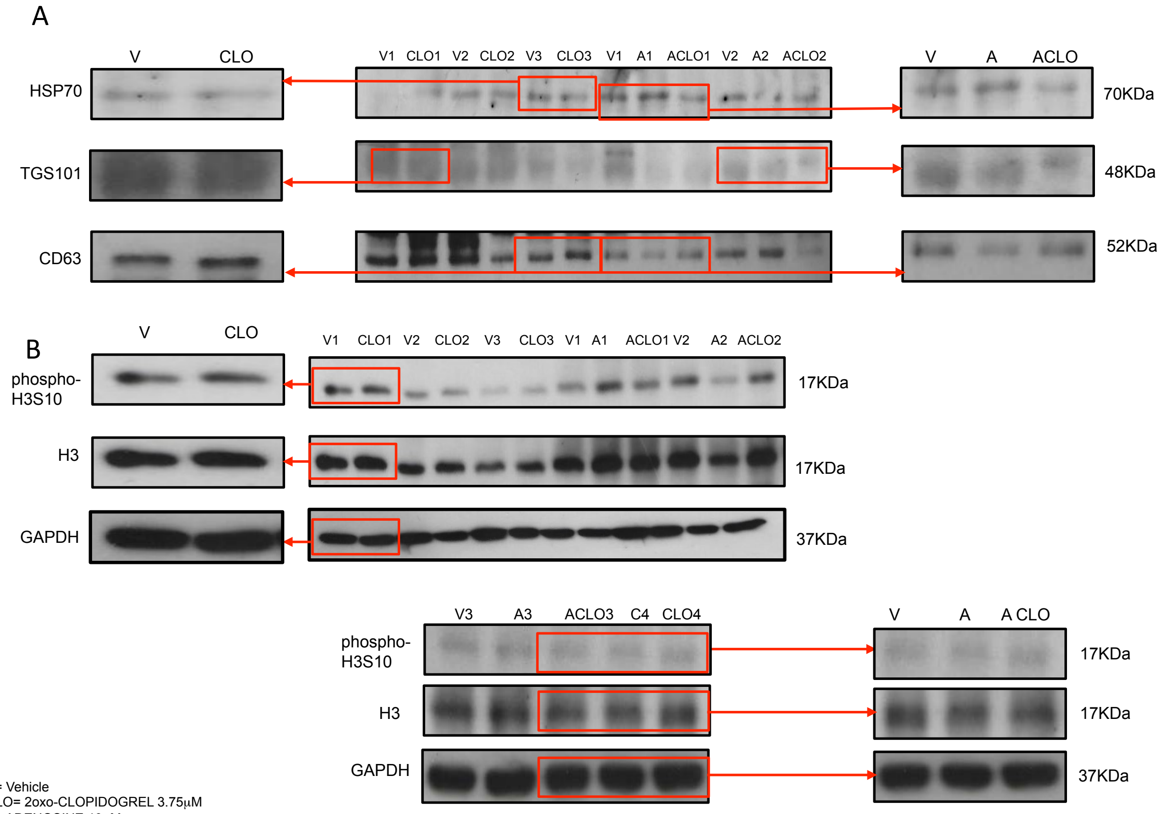

V= Vehicle
CLO= 2oxo-CLOPIDOGREL 3.75μM
A= ADENOSINE 10μM
ACLO= ADENOSINE 10μM+2oxo-CLOPIDOGREL 3.75μM

Supplemental Figure 10.

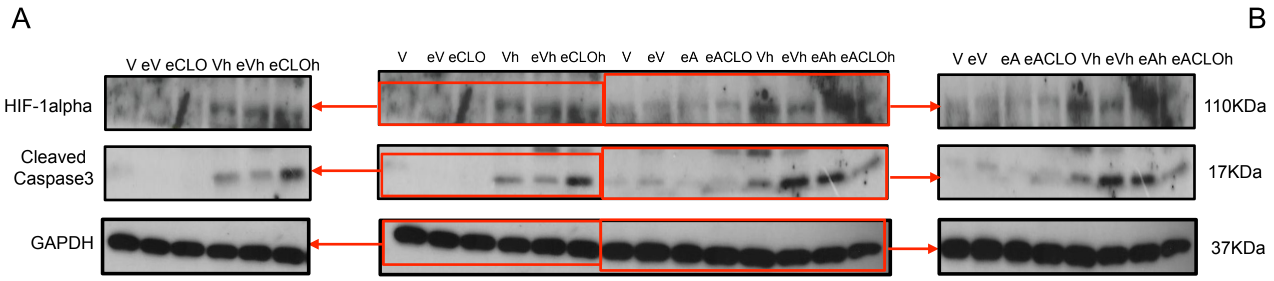

V= Vehicle
eV= exo-VEHICLE
eCLO= exo-2oxo-CLOPIDOGREL 3.75μM
Vh= VEHICLE HYPOXIA
eVh= exo-VEHICLE HYPOXIA
eCLOh= exo-2oxo-CLOPIDOGREL 3.75μM HYPOXIA
eA= exo-ADENOSINE 10μM
eACLO= exo-ADENOSINE10μM+2oxo-CLOPIDOGREL 3.75μM
eAh= exo-ADENOSINE 10μM HYPOXIA
eACLOh= exo-ADENOSINE10μM+2oxo-CLOPIDOGREL 3.75μM HYPOXIA
